# Micro-encapsulated and colonic-release sodium butyrate modulates gut microbiota and improves abdominal pain in patients with symptomatic uncomplicated diverticular disease

**DOI:** 10.3389/fmed.2025.1487892

**Published:** 2025-02-26

**Authors:** Antonio Tursi, Giorgia Procaccianti, Rudi De Bastiani, Silvia Turroni, Federica D’Amico, Leonardo Allegretta, Natale Antonino, Elisabetta Baldi, Carlo Casamassima, Giovanni Casella, Mario Ciuffi, Marco De Bastiani, Lorenzo Lazzarotto, Claudio Licci, Maurizio Mancuso, Antonio Penna, Giuseppe Pranzo, Guido Sanna, Cesare Tosetti, Maria Zamparella, Marcello Picchio

**Affiliations:** ^1^Territorial Gastroenterology Service, Barletta-Andria-Trani Local Health Agency, Andria, Italy; ^2^Department of Medical and Surgical Sciences, School of Medicine, Catholic University, Rome, Italy; ^3^Unit of Microbiome Science and Biotechnology, Department of Pharmacy and Biotechnology, University of Bologna, Bologna, Italy; ^4^GIGA-CP Italian Association for Primary Care Gastroenterology, Feltre, Italy; ^5^Division of Gastroenterology, “Santa Caterina Novella” Hospital, Galatina, Italy; ^6^General Pratictioner, Private Practice Gastroenterologist, Bisceglie, Italy; ^7^General Pratictioner, Private Practice Gastroenterologist, San Ferdinando di Puglia, Italy; ^8^Private Practice Gastroenterologist, Monopoli, Italy; ^9^Private Practice Gastroenterologist, Bari, Italy; ^10^Ambulatory for IBD Treatment, “Valle D’Itria” Hospital, Martina Franca, Italy; ^11^Division of Surgery, “P. Colombo” Hospital, Velletri, Italy

**Keywords:** diverticulosis, symptomatic uncomplicated diverticular disease, gut microbiota, primary care, abdominal pain

## Abstract

The role of gut microbiota (GM) in the pathogenesis of Symptomatic Uncomplicated Diverticular Disease (SUDD) remains controversial. Here, we assessed the efficacy of a butyrate formulation in modulating GM and abdominal pain in patients with SUDD. A retrospective study was conducted in patients with SUDD who were treated with a delayed- and colonic-release formulation of butyrate (two capsules bid, for a total dose of 400 mg butyrate) for 3 months. GM was profiled before (T0) and after 90 days of treatment (T2) using 16S rRNA amplicon sequencing. The primary endpoint was change in GM at T2; secondary endpoints were reduction in abdominal pain severity according to VAS (Visual Analog Scale, 0: absence; 10: maximum severity) at T1 (45 days) and T2, stool characteristics according to the Bristol stool form scale at T0, T1 and T2, and safety of treatment. Fifty-nine patients with SUDD (59.3% male; median age 65.5 years, interquartile range 55–71 years) completed treatment. The butyrate formulation increased GM diversity and resulted in several compositional changes that were closely related to baseline abdominal pain severity. Regarding secondary endpoints, abdominal pain decreased significantly over time, while the Bristol stool form scale did not. Mild adverse events were recorded in 3 (5.08%) patients. This study showed that a microencapsulated and colonic-release formulation of butyrate favorably modulates GM and reduces abdominal pain in patients with SUDD.

## Introduction

Symptomatic Uncomplicated Diverticular Disease (SUDD) is the most common clinical presentation in patients with colonic diverticulosis, with a prevalence of up to 20% (1). Although the pathogenesis of SUDD is not clearly understood, heredity, inflammation, motility alterations and gut microbiota (GM) imbalance (i.e., dysbiosis) are the main pathogenetic factors hypothesized. In particular, the GM of patients with SUDD has been found to be depleted in taxa with anti-inflammatory properties, such as *Clostridium cluster IV*, *Clostridium cluster IX*, *Fusobacterium,* and *Lactobacillaceae* (2), while enriched in mucin-degrading bacteria, such as *Akkermansia muciniphila* (3). More recently, our work using next-generation sequencing has confirmed that specific taxa may be related to SUDD, but the associations vary depending on the severity of abdominal pain (4).

Regarding treatment options, dietary fiber appears to be useful in preventing the onset of colonic diverticulosis, but there is no clear evidence of its benefit in SUDD (5–8). Mesalazine, an intestinal anti-inflammatory drug, may be effective in the symptoms of SUDD and in the prevention of diverticulitis (9), but not in the prevention of recurrence of diverticulitis (10). Probiotics, particularly bifidobacteria, appear to be effective in the prevention of SUDD (3, 11), even in primary care settings (12). Recent studies in general practice have confirmed the high efficacy, safety and excellent tolerability of rifaximin, a non-absorbable enteric antibiotic (12–15). Very often, however, treatment to prevent the symptoms of SUDD is given in a pulsatile manner (7–10 days per month) without scientific evidence (16). Among foods for special purposes, butyric acid may be effective in SUDD by improving gut health in a variety of ways (16, 17). In fact, butyrate is a short-chain fatty acid (SCFA) that serves as an important source of energy for colonocytes, regulates motility, pH and blood flow, improves mucosal barriers, and exerts antioxidant, anti-inflammatory and antimicrobial properties (18). It has been successfully used in the form of sodium butyrate in the treatment of several pathologies affecting the colon, ranging from irritable bowel syndrome (IBS) (19) to inflammatory bowel disease (IBD) (20), but no data are available for SUDD.

Here, we aimed to evaluate the efficacy and safety of a delayed- and colonic-release butyrate formulation in modulating GM and abdominal pain in patients with SUDD treated in primary care. As general practitioners (GPs) often diagnose the disease and request a GM assessment for patients complaining of gastrointestinal symptoms (5, 21), such a study could be helpful for primary care, which is increasingly involved in the management of patients with SUDD.

## Materials and methods

### Study design

We retrospectively assessed the impact of a delayed- and colonic-release butyrate formulation (Butyrose®, SILA Spa) in a population of patients with SUDD managed in primary care by GPs and territorial gastroenterologists. We analyzed stool samples collected by fecal swab for microbiological studies and stored at the Unit of Microbiome Science and Biotechnology, Department of Pharmacy and Biotechnology, University of Bologna (Bologna, Italy). Among them, we identified SUDD patients whose fecal samples were collected before and after treatment with Butyrose® between 1 March 2022 and 1 March 2023. All fecal swabs were collected using the eNAT® System (Copan, Brescia, Italy), and shipped to the Unit of Microbiome Science and Biotechnology, Department of Pharmacy and Biotechnology, University of Bologna (Bologna, Italy), where they were stored at −80°C until processing.

A common database was created to collect the following demographic and clinical data at baseline: gender; age at diagnosis; smoking habit; disease duration; comorbidities; concomitant medications; body mass index; method used to pose the diagnosis of SUDD [colonoscopy, computed tomography (CT), ultrasonography] (1); type of diet followed: Mediterranean diet, predominantly meat-based diet (i.e., more than 5 meals per week based on meat), predominantly fish-based diet (i.e., more than 5 meals per week based on fish), predominantly plant-based diet (i.e., more than 5 meals per week based on fruit and vegetables), vegetarian diet [i.e., a diet that excludes meat (fresh or processed, including cured meats) and fish, but includes the consumption of animal products such as dairy products, eggs and honey] (22, 23), vegan diet, i.e., a diet that excludes all foods of animal origin, such as meat and fish, but also dairy products, eggs and honey (22, 24). The severity of abdominal pain was measured using a 10-point visual analog scale (VAS) before treatment (T0), after 45 days of treatment and at the end of treatment (T2).

The study was conducted according to clinical practice guidelines and following the principles of the Declaration of Helsinki. All patients gave written informed consent before undergoing endoscopy and/or CT scan and/or fecal sampling. Ethic committee approval for this retrospective study was obtained from Azienda Ospedaliero-Universitaria “Ospedali Riuniti,” Foggia, Italy (PROT. 164/CE/2023, October 23, 2023).

### Inclusion criteria

Inclusion criteria were: males and females aged >18 years; colonic diverticulosis diagnosed by colonoscopy or imaging (abdominal CT scan and/or ultrasonography); diagnosis of SUDD (defined as left-lower and long-lasting quadrant pain in patients with diverticulosis) (25) during the 6 months prior to enrolment; possibility of retrospectively reconstructing the symptoms and clinical history of patients with SUDD; availability of a fecal sample before (T0) and after (T2) 90 days of Butyrose® supplementation for GM assessment (see also “Primary endpoint”).

### Exclusion criteria

Exclusion criteria were: current or previous diagnosis (by abdominal CT and/or ultrasonography) of acute diverticulitis (defined as inflammation of the colonic wall harboring diverticula with fat stranding, with or without complications such as abscesses, stenosis or fistulas, namely uncomplicated or complicated diverticulitis) (1); IBD; ischemic colitis; prior colonic resection; patients with severe liver failure (Child-Pugh C); patients with severe kidney failure; pregnant women; women of childbearing potential not using a highly effective method of contraception; patients currently using or who have received any laxative agents <4 weeks prior to enrolment; patients currently using or who have received any mesalamine compounds <4 weeks prior to enrolment; patients currently using or who have received any probiotic agents <4 weeks prior to enrolment; use of non-steroidal anti-inflammatory drugs (NSAIDs; except for acetyl-salicylic acid ≤100 mg/day) <4 weeks prior to enrolment; patients treated with antibiotics (including those not absorbed) <4 weeks prior to enrolment; patients with a history of cancer, of any origin, at the time of SUDD diagnosis and/or under treatment with chemotherapy and/or radiotherapy; a history of alcohol, drug, or chemical abuse; patients with a current or recent (≤3 months) episode of COVID-19 (26).

### Treatment and product

As noted above, only patients taking Butyrose® (two tablets per day, 1 after lunch and 1 after dinner, for a total of 1,100 mg of sodium butyrate per day) as mono-therapy for 3 months were enrolled. Butyrose® is a micro-encapsulated sodium butyrate-based supplement manufactured with a modified-release LSC MicroCaps® delivery mechanism (EU patent 2,352,386) for exclusive release in the colon. This formulation was studied because sodium butyrate is a very active molecule but has a high degree of dissociation (pKa 4.82) and would not reach the colon/rectum without an adequate delayed-release mechanism. Butyrose® is manufactured and marketed by SILA s.p.a. (Noale, VE, Italy) and its marketing was notified to the Italian Regulatory Authorities in October 2021.

### GM profiling using 16S rRNA amplicon sequencing

#### a. Microbial DNA extraction

Fecal samples were processed as described by Tursi et al. (4). Briefly, swabs were vortexed and centrifuged at 13,000 rpm for 10 min at 4°C. The pellets were resuspended in 1 mL of lysis buffer (500 mM NaCl, 50 mM Tris–HCl pH 8, 50 mM EDTA, and 4% SDS) and subjected to three rounds of bead-beating in a FastPrep instrument (MP Biomedicals, Irvine, CA, United States) at 5.5 movements/s for 1 min, in the presence of four 3-mm glass beads and 0.5 g of 0.1-mm zirconia beads (BioSpec Products, Bartlesville, OK, United States). After incubation at 95°C for 15 min, the samples were centrifuged at 13,000 rpm for 5 min, and the supernatants were added with 260 μL of 10 M ammonium acetate for protein precipitation. After centrifugation at 13,000 rpm for 10 min, nucleic acids were precipitated with isopropanol, and the pellets were washed with 70% ethanol before being resuspended in 100 μL of TE buffer (10 mM Tris–HCl, 1 mM EDTA, pH 8.0). After treatment with 2 μL of 10 mg/mL DNase-free RNase at 37°C for 15 min, DNA was purified using the DNeasy Blood and Tissue Kit (QIAGEN, Hilden, Germany) according to the manufacturer’s instructions. DNA quantity and quality were assessed using a NanoDrop ND-1000 spectrophotometer (NanoDrop Technologies, Wilmington, DE, United States).

#### b. 16S rRNA gene amplification and sequencing

The V3-V4 hypervariable regions of the 16S rRNA gene were amplified using the 341F and 785R primers with Illumina adapter overhangs as previously described (27). After amplicon purification using a magnetic bead-based clean-up system (Agencourt AMPure XP, Beckman Coulter, Brea, CA, United States), indexed libraries were prepared by limited-cycle PCR using Nextera technology, further purified and pooled at an equimolar concentration of 4 nM. The pool was denatured and diluted to 5 pM prior to sequencing on an Illumina MiSeq platform (Illumina, San Diego, CA, United States) using a 2 × 250 bp paired-end protocol. Raw sequencing data were deposited in the National Center for Biotechnology Information Sequence Read Archive (BioProject ID: PRJNA1216941).

#### c. Bioinformatics

Raw sequences were processed using PANDASeq (28) and QIIME 2 (29) and filtered for length and quality. Amplicon sequence variants (ASVs) were identified using DADA2 (30) and taxonomically classified using the VSEARCH algorithm (31) against the SILVA database (August 2020 release) (32). Alpha diversity was calculated using various metrics, such as the number of observed ASVs, the Shannon index and Faith’s phylogenetic diversity. Beta diversity was calculated using UniFrac distances, which were used to construct Principal Coordinates Analysis (PCoA) plots.

### Primary endpoint

We retrospectively evaluated the impact of a butyrate formulation (Butyrose®) on the GM of a cohort of patients with SUDD. GM was assessed using next-generation sequencing of fecal samples collected before (T0) and after (T2) 90 days of treatment.

### Secondary endpoints

The following secondary endpoints were evaluated:

- Efficacy of the butyrate formulation (Butyrose®) in reducing abdominal pain in patients with SUDD, defined as at least a 50% reduction in abdominal pain, assessed at T0, T1 (after 45 days of treatment) and T2 using VAS (0: absence; 10: maximum severity);
- Characteristics of evacuation according to the Bristol stool form scale (33) at T0, T1 and T2;
- Safety of therapy, assessed as the number and type of adverse events observed during the 3 months of treatment.

### Statistical analysis

All statistical analyses were performed using R software and the vegan^1^ and Made4 (34) packages. GM data separation in PCoA was tested using a permutation test with pseudo-F ratio (PERMANOVA). Pre-post differences in alpha diversity, relative taxon abundance and abdominal pain were assessed using the Wilcoxon signed-rank test. The chi-square test was used to compare pre-post differences in Bristol stool form scale. *p*-values were adjusted for multiple comparisons using the Benjamin-Hochberg method, with a false discovery rate (FDR) ≤0.05 considered statistically significant.

## Results

### Study cohort description

According to the inclusion and exclusion criteria, 72 patients with SUDD treated with Butyrose® were identified. Of these, 59 patients completed the 3 months of treatment, while 13 did not and were excluded from the final evaluation: 4 patients were lost at follow-up, 1 developed a urinary tract infection, 1 had a recurrence of SUDD, 1 developed acute uncomplicated diverticulitis, and 6 received adjunctive treatment for symptom control (2 with mesalazine, 3 with rifaximin, and 1 with mesalazine plus rifaximin). Demographic and clinical characteristics of the patients are reported in Table 1.

**Table 1:** Demographic and clinical characteristics of patients with SUDD at enrolment.

	Study group (*n* = 59)
Male gender, n (%)	35 (59.3)
Median (IQR) age, years	65.5 (55–71)
Body mass index, median (IQR) kg/m^2^	26.0 (24–29)
Symptom duration, median (IQR), months	3 (1–4)
Presence of comorbidities, n (%)	47 (79.7)
Cardiovascular	22 (46.8)
Metabolic	11 (23.4)
Respiratory	5 (10.6)
Rheumatic	3 (6.4)
Others	6 (12.8)
Previous appendectomy, n (%)	8 (13.5)
Diagnostic tool, n (%)	
Colonoscopy	44 (74.6)
Computed tomography	5 (8.5)
Ultrasonography	10 (16.9)
Diet, n (%)	
Mediterranean	42 (71.2)
Prevalence of meat	7 (11.9)
Prevalence of fish	–
Vegetarian	10 (16.9)
Vegan	–
Abdominal pain, median (IQR) VAS score	5 (3–10)
Bristol stool form scale, median (IQR)	4 (3–5)

IQR, interquartile range; VAS, visual analog scale.

### Primary endpoint

Fecal samples were collected from 59 SUDD patients before and after 90 days of taking Butyrose® and analyzed for GM changes.

#### a. Impact of Butyrose® on the gut microbiota of patients with SUDD

**Figure 1:**
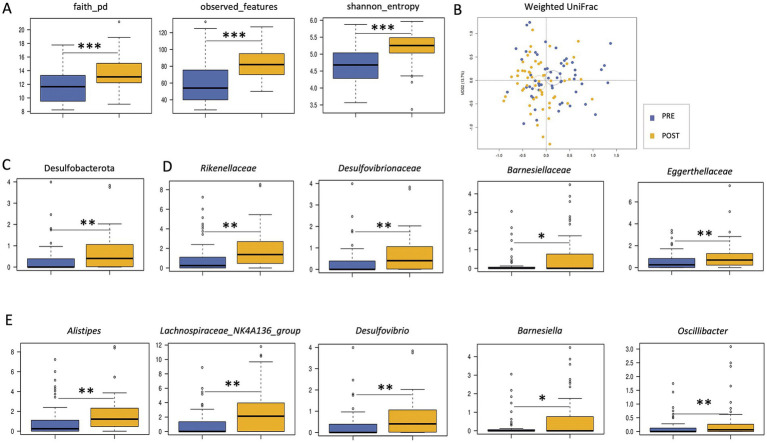
Impact of Butyrose® on the gut microbiota of SUDD patients. **(A)** Boxplots showing the distribution of alpha diversity, computed according to Faith’s phylogenetic diversity, number of observed ASVs and Shannon entropy, in the gut microbiota of SUDD patients before (PRE) and after (POST) 90 days of Butyrose® supplementation. **(B)** Principal Coordinates Analysis (PCoA) based on weighted UniFrac distances between groups. Ellipses include 95% confidence area based on the standard error of the weighted average of sample coordinates. A significant separation was found (PERMANOVA, *p* = 0.001). Boxplots showing the relative abundance distribution of phyla **(C)**, families **(D)** and genera **(E)** differentially represented between groups. Wilcoxon test, *p ≤ 0.05; **p ≤ 0.01; ***p ≤ 0.001.

A significant increase in alpha diversity was observed after treatment (Wilcoxon test, *p* < 0.001; Figure 1A). Similarly, PCoA based on weighted UniFrac distances showed a significant separation before and after treatment (PERMANOVA, *p* = 0.001; Figure 1B). From the taxonomic point of view, many differences emerged. In particular, the phylum Desulfobacterota, the families *Eggerthellaceae, Rikenellaceae* (and its genus *Alistipes*)*, Barnesiellaceae* (and *Barnesiella*) and *Desulfovibrionaceae* (and *Desulfovibrio*), and the genera *Lachnospiraceae_NK4A136_group* and *Oscillibacter* were enriched after treatment (Wilcoxon test, *p* < 0.05; Figures 1C–E).

#### b. Impact of Butyrose® on the gut microbiota according to severity of abdominal pain

**Figure 2:**
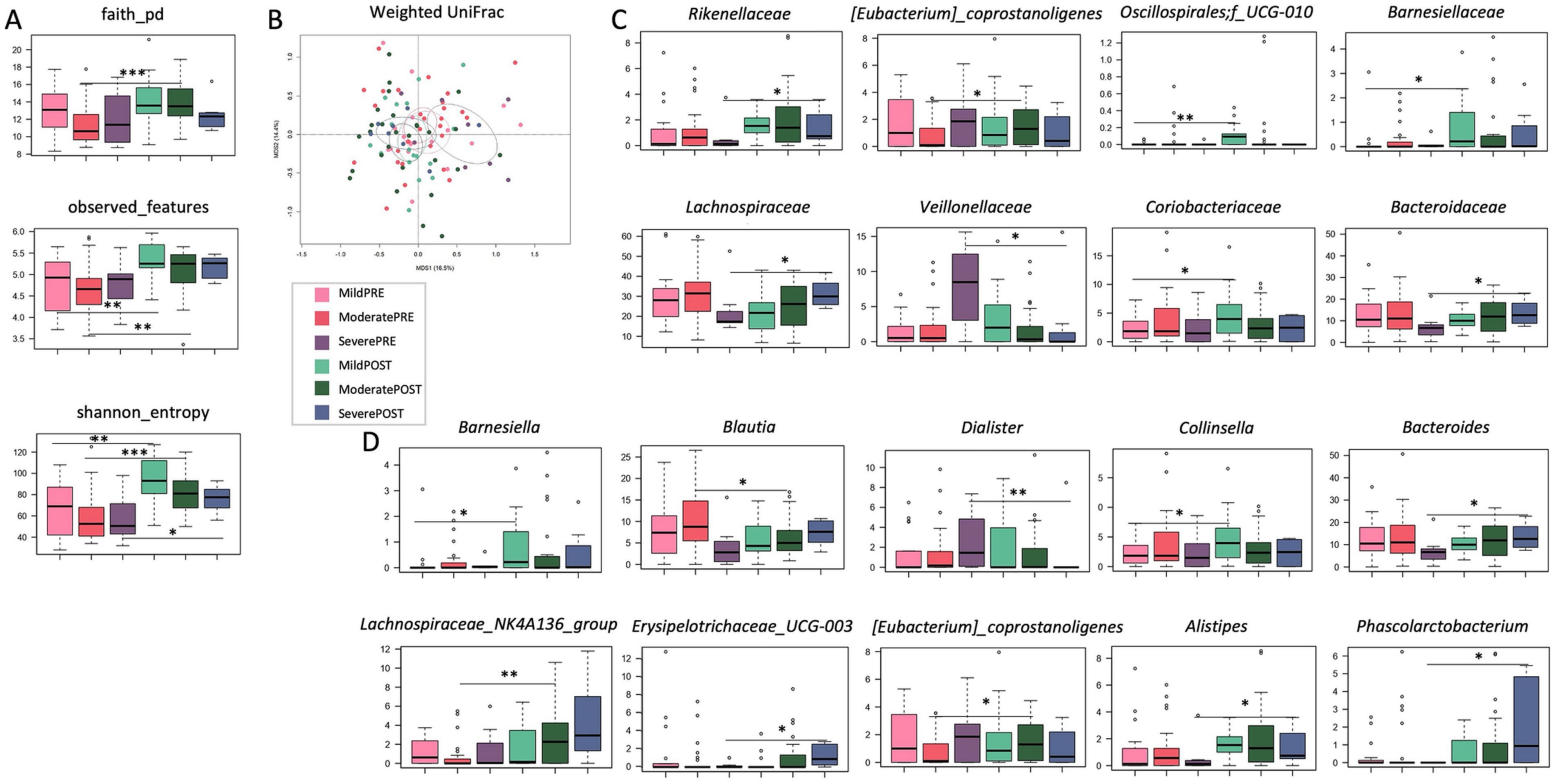
Effects of Butyrose® according to basal abdominal pain severity. **(A)** Boxplots showing the distribution of alpha diversity, computed according to Faith’s phylogenetic diversity, number of observed ASVs and Shannon entropy, in the gut microbiota of SUDD patients stratified by abdominal pain severity [estimated by visual analog scale, VAS: mild (VAS score 1–3) vs. moderate (VAS score 4–7) vs. severe (VAS score 8–10)] before (PRE) and after (POST) 90 days of Butyrose® supplementation. **(B)** Principal Coordinates Analysis (PCoA) based on weighted UniFrac distances between groups. A significant separation was found (PERMANOVA, *p* = 0.019). Boxplots showing the relative abundance distribution of families **(C)** and genera **(D)** differentially represented between groups. Wilcoxon test, *p ≤ 0.05; **p ≤ 0.01; ***p ≤ 0.001.

SUDD patients were stratified by basal abdominal pain severity [mild (VAS score 1–3) vs. moderate (VAS score 4–7) vs. severe (VAS score 8–10)] and all analyses were repeated. The treatment-related increase in alpha diversity was confirmed in all severity groups (Wilcoxon test, *p* < 0.01; Figure 2A). For beta diversity, a significant segregation among groups was observed in the weighted UniFrac-based PCoA (PERMANOVA, *p* = 0.019; Figure 2B). Taxonomically, none of the differences observed in the overall cohort were replicated in all groups, suggesting that the impact of Butyrose® was dependent on basal abdominal pain severity. In particular, the increase in the family *Rikenellaceae* (and its genus *Alistipes*) was significant only in the severe group, the increase in *Barnesiellaceae* (and *Barnesiella*) only in the mild group, and the increase in *Lachnospiraceae_NK4A136_group* only in the moderate group (Wilcoxon test, *p* < 0.05; Figures 2C,D). Furthermore, the mild group showed enrichment in *Coriobacteriaceae* (and *Collinsella*) and *Oscillospirales_UCG-010* (p < 0.05). The moderate group showed enrichment in *[Eubacterium] coprostanoligenes_group* and depletion in *Blautia* (*p* < 0.05). Finally, the severe group showed enrichment in *Lachnospiraceae*, *Bacteroidaceae* (and *Bacteroides*)*, Erysipelotrichaceae_UCG-003* and *Phascolarctobacterium*, and depletion in *Veillonellaceae* (and *Dialister*; *p* < 0.05).

### Secondary endpoints

**Figure 3:**
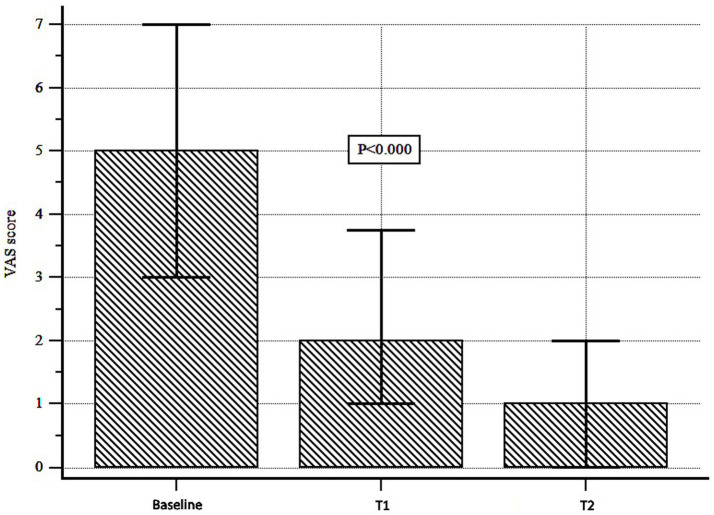
Abdominal pain assessment during Butyrose® treatment. Abdominal pain was assessed by visual analog scale (VAS). A significant reduction was observed over time (Wilcoxon test, *p* < 0.000).

Abdominal pain decreased significantly over time [median (interquartile range, IQR) VAS score: 5 (3–10) at T0, 2 (1–3.7) at T1 and 1 (0–2) at T2; Wilcoxon test, *p* < 0.000; Figure 3]. The Bristol stool form scale did not change significantly over time (*p* = 0.996; Table 2). Adverse events were recorded in 3 patients (5.08%): they were mild (1 patient complained of nausea and 2 of diarrhea), and did not require discontinuation. Notably, all these events occurred during a concurrent epidemic of viral gastroenteritis.

**Table 2:** Bristol stool form scale at baseline and during Butyrose® treatment.

Type	Baseline	T1	T2
1. Separate hard lumps, like nuts (difficult to pass).	6 (10.2)	5 (8.5)	6 (10.2)
2. Sausage-shaped, but lumpy.	7 (11.9)	10 (16.9)	7 (11.9)
3. Like a sausage but with cracks on its surface.	14 (23.7)	12 (20.3)	15 (25.4)
4. Like a sausage or snake, smooth and soft (average stool).	13 (22.0)	18 (30.5)	16 (27.1)
5. Soft blobs with clear cut edges.	9 (15.2)	7 (11.9)	7 (11.9)
6. Fluffy pieces with ragged edges, a mushy stool (diarrhea).	7 (11.9)	5 (8.5)	6 (10.2)
7. Watery, no solid pieces, entirely liquid (diarrhea).	3 (5.1)	2 (3.4)	2 (3.4)

Values are expressed as number (percentage of patients). *p* = 0.996, Chi-square test.

## Discussion

Here, we investigated the effects of a butyrate formulation (Butyrose®) on GM in patients with SUDD. Moreover, we investigated the effect of this formulation on abdominal pain and its association with GM.

With regard to the primary endpoint, namely the impact of Butyrose® on GM, we found that treatment resulted in an increase in alpha diversity, the reduction of which is a typical hallmark of dysbiosis in a variety of diseases, both local and systemic (35). At the compositional level, the patients’ GM was found to be enriched in several taxa after treatment, including *Desulfovibrio* (and its family *Desulfovibrionaceae* and its phylum Desulfobacterota), *Alistipes* (and its family *Rikenellaceae*), *Barnesiella* (and *Barnesiellaceae*), *Eggerthellaceae*, *Lachnospiraceae_NK4A136_group* and *Oscillibacter*. In particular, *Desulfovibrio* belongs to sulfate-reducing bacteria, which are considered pathobionts that may contribute to intestinal and extra-intestinal diseases, probably through the production of hydrogen sulfide, lipopolysaccharide and mucolytic enzymes, and the secretion of outer membrane vesicles (36). Although apparently unfavorable, the increase in sulfate-reducing bacteria may be related to their ability to use various organic compounds, including butyric acid, as electron donors for sulfate reduction (37–39). It should be noted that some *Desulfovibrio* species/strains appear to have more pathogenic potential than others, particularly in unhealthy individuals (36), highlighting the need for high-resolution taxonomic profiling and in-depth assessment of context-dependent features, including interaction networks within the microbial community and with the host. Another potentially harmful genus enriched after treatment was *Alistipes*, a bile-tolerant microorganism that has been shown to contribute to some diseases but also to protect against others (40), again emphasizing the need for further studies, including animal models, to better understand its role in health and disease. On the other hand, the increases in the SCFA producers *Oscillibacter* and *Lachnospiraceae_NK4A136_group* can certainly be considered positive, in addition to being consistent with the available literature on the effects of Butyrose® on the GM of patients with IBD (41). In particular, *Lachnospiraceae_NK4A136_group* has been shown to have protective and anti-inflammatory effects, including improvement of gut barrier function (42–44). *Oscillibacter*, together with *Alistipes* species, has recently been found to be enriched in subjects with lower plasma triglycerides and glucose and higher plasma HDL in ethnically distinct cohorts (45), suggesting a role in cardiovascular health, the impairment of which may be related to SUDD (46).

Based on our previous work showing that the GM of SUDD patients stratified by severity of abdominal pain (as assessed by VAS) (4), we next investigated the effects of Butyrose® in patients with mild vs. moderate vs. severe SUDD. While the increase in alpha diversity was common to all severity groups, the compositional variations were closely related to baseline severity (of abdominal pain and dysbiosis), with the severe group showing an overall greater rearrangement compared to the other groups. In particular, the severe group showed an enrichment in *Rikenellaceae* (and *Alistipes*), *Lachnospiraceae*, *Bacteroidaceae* (and *Bacteroides*)*, Erysipelotrichaceae_UCG-003* and *Phascolarctobacterium*, and a depletion in *Veillonellaceae* (and *Dialister*) after treatment. Again, some changes could be beneficial, such as those in the SCFA producers *Lachnospiraceae* and *Phascolarctobacterium* (47), while others could not, notably that in *Erysipelotrichaceae_UCG-003*, which has previously been linked to intestinal dysfunction, inflammation and metabolic disorders (48). Conversely, the other severity groups showed fewer changes, in line with the lower extent of their dysbiosis, namely an enrichment in *Lachnospiraceae_NK4A136_group* and *[Eubacterium] coprostanoligenes_*group and a depletion in *Blautia* in the moderate group, and an enrichment in *Barnesiellaceae* (and *Barnesiella*), *Coriobacteriaceae* (and *Collinsella*) and Oscillospirales_UCG-010 in the mild group. Among these, it is worth noting that *Blautia* has previously been positively associated with gastrointestinal symptoms (particularly diarrhea) and IBS (49), making its reduction potentially desirable also in the context of SUDD. On the other hand, the increase in *Collinsella* is again questionable, as this genus has been negatively associated with IBS severity (50), but is also known as a pathobiont capable of increasing gut permeability and triggering pro-inflammatory cytokines (51, 52).

With regard to secondary endpoints, we found that Butyrose® was effective in controlling abdominal pain, the main symptom characterizing SUDD patients. This was expected as butyrate is a SCFA that, among other effects, improves gut permeability, increases the rate of cell regeneration, reduces oxidative stress and mucosal inflammation (18), and has previously been shown to be reduced in stool samples from patients with SUDD (4, 5). In contrast, no changes in stool appearance were recorded under treatment with Butyrose®. This may mean that other factors, such as colonic motility, are involved in determining stool form in these patients and/or that the type of fecal output did not influence the clinical response to butyrate. Importantly, however, this means that Butyrose® does not cause constipation, an adverse event that has often been hypothesized but never confirmed with older formulations of butyrate (53). As for adverse events, their incidence was quite low in our population (~5%). However, it is not easy to explain this occurrence, in particular for patients experiencing diarrhea. In fact, sodium butyrate is recommended for the treatment of diarrheal disorders such as traveler’s diarrhea (54). However, we are not sure that these adverse events could really be related to butyrate, as they all occurred during a concurrent epidemic of viral gastroenteritis.

The main limitations of the study include: (i) the use of 16S rRNA amplicon sequencing, which is still the gold standard for microbiota profiling, but does not allow high-resolution taxonomic profiling down to species level and functional insights; (ii) the lack of mechanistic information; and (iii) the retrospective design. In particular, the retrospective design may have led to the loss of information that could have influenced the final results (e.g., recall of symptom severity may not have been reliable, and GM-associated confounding factors, such as proton pump inhibitor use, may not have been captured).

In conclusion, Butyrose® supplementation for 90 days significantly modulated the GM of patients with SUDD. Specifically, it led to increased diversity and some compositional changes that were closely related to baseline abdominal pain severity. Some changes may be beneficial, such as the increase in SCFA producers, while others may not be, such as the increase in taxa with dubious pathogenic potential. Regardless of the significance of the increase in these taxa, these changes in GM were associated with a significant improvement in abdominal pain. Further studies in larger cohorts with longer observation periods and using other omics, such as metagenomics and metabolomics, are needed to validate our findings and to gain deeper insights into the impact of Butyrose® on SUDD, including the composition (down to species level, including interaction networks) and function of the GM. Such studies could pave the way for personalized intervention strategies based on disease severity for faster and more effective resolution of symptoms.

## Data Availability

The raw sequencing data of this study were deposited in the National Center for Biotechnology Information Sequence Read Archive (BioProject ID: PRJNA1216941) https://www.ncbi.nlm.nih.gov/bioproject/?term=PRJNA1216941.

## Footnotes

^1^https://cran.r-project.org/package=vegan
